# A desire to be embraced - the lived experience of encountering primary health care for a person with mental health problems. A descriptive phenomenological study

**DOI:** 10.1080/02813432.2025.2587543

**Published:** 2025-11-11

**Authors:** Emmy Nilsson, Lina Behm, Suzanne Johanson, Ulrika Bejerholm

**Affiliations:** ^a^ Mental Health, Activity and Participation, Department of Health Sciences, Lund University, Lund, Sweden; ^b^ Centre of Evidence-based Psychosocial Interventions, CEPI, Lund University, Lund, Sweden; ^c^ Department of Nursing and Integrated Health Sciences, Kristianstad University, Kristianstad, Sweden; ^d^ Section for Health and Rehabilitation, Department of Neuroscience and physiology, Section for health and rehabilitation, Sahlgrenska Academy, Gothenburg University, Gothenburg, Sweden; ^e^ Department of Psychiatry, Region Skåne University Hospital, Lund, Sweden

**Keywords:** Mental health problems, primary health care, phenomenology, person-centered care, lived experience

## Abstract

**Background:**

Primary health care is in the unique position of being a first level of support and care to individuals with mental health problems. The focus in this service is on diagnosis, medical treatment, and symptom reduction. However, to access it is perceived as challenging by both patients and providers. An increased understanding of the lived experience of encountering primary health care may be beneficial for the delivery of a tailored mental health service to patients.

**Aim:**

To explore the lived experience of encounters with primary health care of a person with mental health problems.

**Methods:**

Eleven in-depth interviews were conducted online between October 2022 and April 2023. A descriptive phenomenology study in accordance with Giorgi was used to analyze the material.

**Results:**

The essence of the lived experience of being a patient with MHP was a *desire to be embraced* by health professionals, which was the general construction based on four themes, *To come from a place of loneliness and vulnerability*, *To sense mental health was viewed as problematic*, *To not be in control and To feel safe.*

**Conclusions:**

The lived experience of being a patient with mental health problems was described as everyday challenges due to their mental health. They never knew whether the support was there for them as patients when encountering primary health care. Acknowledging patients as experts on their life situation is the core element in person-centered care. It is therefore crucial for further research to include patients’ experiential knowledge to inform clinical practice and to improve clinical outcomes.

## Introduction

Mental health problems (MHP) are a growing global health concern [1]. They comprise a broad concept encompassing many aspects, from challenges experienced in everyday life to having complex MHPs [2]. There has been a general increase in self-reported MHPs in Northern of Europe [3] and within the Swedish population during the last decades [4]. Having signs and symptoms of MHP are common reasons for seeking primary health care (PHC) [5] and psychiatric diagnoses are one of the three most reported diagnostic criteria [6,7]. However, MHPs are often masked with physical signs and symptoms and may co-occur and co-exist with other illnesses [7–10]. This ambiguous situation makes it difficult for patients to deal with and can lead to a person not knowing when to seek health care [11]. Thus, providing access and maintaining PHC to individuals with MHPs is perceived as challenging by both patients and providers [12–14].

In Sweden, PHC is responsible for preventing, assessing, treating, and rehabilitating patients with health care needs that do not require specialised medical or technical resources, which are only available in secondary or specialised care [15]. Therefore, PHC is in a unique position to provide support for a person with MHPs, being the first level of mental health service to support, manage and ease suffering. Various attempts to integrate services to individuals with MHPs in PHC have been made, thus lowering the threshold for a person with MHPs to access and receive adequate care [16,17]. Furthermore, the implementation of care managers in some PHC settings has contributed to improvements of mental health encounters [18]. Being a care manager has enabled the role of nurses in PHC settings, such as being the patient’s advocacy and safeguarding the patients’ needs for continuity of care. In addition, having a care manager has shown to improve access to care among individuals with MHP [19]. Thus accessibiliy, building caring relationships and being able to meet diverse needs are important aspects in the delivery of qaulity care [20]. To integrate mental health services into PHC has further contributed to the adaptation of a more holistic approach where seeing the person behind the diagnosis is fundamental [21]. However, health professionals’ negative attitudes and stigma towards mental illness [23,24], lack of knowledge, skills, and motivation for change, as well as insufficient management, leadership, resources, and strategies hinder this integration of services [14,19,22,25]. Therefore, the availability of and access to mental health services of good quality still varies considerably across PHC [12,20,22]. Moreover, the non-utilization of individuals’ experiential knowledge about being a patient i.e. referred to a person with MHPs within the physical confines of a health care setting, has also been reported as a barrier during the implementation process of integrated services [26]. Much focus is still on improving existing traditional and medical models rather than on providing holistic, person-centered and tailored mental health care based on the individual preferences, interests, and needs of the person with MHP [1,19,27,28]. Nilsson et al. [22] further showed that health professionals’ mental health literacy, i.e. knowledge, attitude, views, and beliefs about mental health [29], and the organisational capacity of PHC of dealing with MHPs where decisive for the caring actions made. The access to quality care may thus partly be explained by health professionals having a low level of mental health literacy [22,30,31]. Moreover, professionals who have been exposed to a person with MHP [32] or have experiences of building trusting relationship with this group of patients showed fewer negative attitudes towards them [22]. Research shows that they lack knowledge about the lived experience of patients with MHP, what their mental health needs are, and how they perceive meaningful care within the context of PHC [14,24,33]. By this logic, stigmatizing attitudes among health professionals towards individuals with MHP may be altered by exposing them to research knowledge about the lived experiences of MHP [30,34]. The involvement of experiential knowledge in the development of care has been maintained as fundamental, which entails that those who are most affected by the supposed problem should be involved in finding solutions for clinical practice [26,34].

Individuals with MHP frequently describe public stigma and have experience of disparaging attitudes among professionals when describing negative experiences of PHC [35–37]. This was experienced as ‘hiding behind a mask’ [38] and explained as a way for patients to cope with the discomfort of seeking support for their worry, anxiety, or other signs and symptoms of MHPs. Being a person with complex MHP is described as having a devalued view of oneself [39]. A person with MHP is likely in this sense to internalize public stigma [40] and thus self-stigma becomes an obstacle for disclosing their MHPs and getting access to successful treatment and recovery [12,41]. Furthermore, a sociodemographic factor such as gender has been found to be associated with seeking help at PHC for MHPs, where men are more prone not to do this in comparison to women [36]. Furthermore, a person with a lower educational background is less likely to seek health care for their MHPs compared to those with secondary high school education. Reasons for not seeking health care have been found to be awaiting recovery (59%), negative perceptions of health care (34%), ignorance (29%), stigma (23%), and structural barriers (12%) [36]. Early identification and care are often lacking and individuals with MHP express a fear of encountering negative attitudes and stigmatizing views when seeking care and describe a vulnerability and loss of autonomy [12,42]. As emphasized in previous research, a trusting relationship and a tailored integrated care are critical for how a person with MHPs perceives the care received [12,19–21,27,42,43]. Active listening and trying to understand patients’ perceived health are two examples of how health professionals may support early recognition of MHP, including routine screening for suicide attempts [22,42,44].

Altogether, the lived experiences of a person with MHP as they encounter PHC are critical to explore in research. Such experiential knowledge is stated as a prerequisite for the understanding of person-centered practice [2,28] and could possibly inform professionals to recognize and support an open and co-creative dialogue about mental health in PHC [22,27,45]. A greater understanding may thus benefit PHC delivery and thus outcomes for a person with MHPs [27]. PHC that make use of experiential knowledge may even reduce the inequalities patients with MHP describe [20,27,45]. To our knowledge, however, experiential knowledge is lacking in a Swedish PHC context, exploring their concerns, expectations and what is essential to them when in contact with PHC.

## Aim

To explore the lived experience of encounters with primary health care of a person with mental health problems.

## Methods

### Study design

A descriptive phenomenological study design in accordance with Giorgi [46] was chosen to meet the study aim. The approach is grounded on Husserl’s philosophy of phenomenology. In this view, the meaning of a person’s lifeworld emerges when their consciousness focuses on a particular experience. This experience, or phenomenon, is shaped by how participants describe it in their narratives. They will generate different understandings of how the phenomenon is presented to them.

### Context and setting

The research context was PHC, which is the first level of mental health service for a person with MHP. This context consisted of regional PHC centers and child health centers, student welfare and municipality health care such as school health services and elderly care. A criterion sampling procedure was applied [47] and the inclusion criteria were: a person with perceived MHPs, lived experience of being a patient encountering PHC for their MHPs <5 years, >16 years of age, able to communicate in Swedish, and cognitively lucid, i.e. having provided both written and verbal informed consent to participate, being aware of the aim of the study, and being able to proceeded with the interviews. To reach out to potential participants, termed patients from here on, an advertisement with information about the study and inclusion criteria was created. A picture was also inserted which reflected the mental health field and PHC. To attract young adult patients, who are the group with the highest prevalence of MHPs, young adults in a school setting were asked to provide verbal and written feedback on four pre-chosen pictures to inform the choice of picture to be used in the advertisement. This was then digitally distributed on social media platforms of user organizations and research network platforms or printed on posters to be put up on information boards at PHC centers. The advertisement was also linked to a webpage at Lund University [LU] where potential patients could register their interest in participating. They were then contacted *via* an ‘Expression of interest e-mail’ with pre-formulated information about the study aim, and a question whether they still were interested. If this was the case, they could reply with a signed consent form. Fourteen patients with lived experience of being a patient in PHC were enrolled in the study and an interview time was scheduled for each of them. A reminder e-mail was sent to those who had not responded within three weeks. Eleven patients responded and gave their written consent to participate. Two patients did not respond, and one declined participation after receiving initial information due to limited time for participation.

### Data collection

All researchers applied an open attitude towards the phenomenon, where previous knowledge was set aside and the description of the person is understood to be what they were presented to, their consciousness of the phenomenon was taken as presences. The research group included a wide range of experience from being a novice researcher within the field of PHC and mental health, to researchers within a multi-disciplinary research group and national research network context within the field of mental health, user involvement and co-production. The experienced knowledge also spans from lived experiences of signs and symptoms of MHP to work as health professionals with patients with MHPs in a PHC or mental health service context. An interview guide inspired by Giorgi [46], Matua [48], and Robinson & Englander [49] were applied, it included open-ended questions to capture rich data with an open attitude to why, when and what the phenomenon meant and how it presented itself to the patients with lived experience of PHC, see Table 1. Individual in-depth interviews were conducted from October 2022 to April 2023, primarily by the first author (EN) and secondly by the third author (SJ). The interviews took place on a secure LU-Zoom platform, using a password and managed on servers within the European Union. The interviews lasted between 34 to 80 min (median 43 min) and were audio recorded and then transcribed word by word. Socio-demographic characteristics of the participants were collected to support the data analysis. The enrolment period lasted for six months. A consensus discussion of the first interviews was conducted among all authors with a focus on being open to the phenomenon (putting our own knowledge and preunderstanding on hold). A critical discussion of the relevance of the probing questions was key to capturing the narrative of the person’s lived experience [46]. No additional questions were added to the interview guide.

**Table 1.** Intervjuguide [interview guide].

**Tema** *Vad hände, hur hände det och hur har det påverkat personen, utifrån Matua (2015).* *[What occurred, how it occurred, and the impact it had on the individual as proposed by Matua (2015)]*
**Huvudfråga:** [Main question]
**Vad är din erfarenhet av att söka vård inom primärvården för din psykiska hälsa?** *[What is your experience of seeking care within primary health services for your mental health?]*
**Uppföljningsfrågor:** [Follow up questions]
**1. Vad hände?** [What happened/occurred]
**Vill du beskriva ditt senaste möte för din psykiska hälsa inom primärvården?** [Would you like to describe by your lived experience your last encounter with PHC for your mental health] Vad var positivt? [What was positive] Vad var negativt? [What was negative]
**Kan du beskriva vilket stöd eller planering som erbjöds vid ditt senaste möte?** [Can you describe which support or care plan that was being offered at your last encounter]
**Vill du beskriva hur din kunskap om din egen hälsa togs till vara på av vårdpersonalen vid det senaste mötet?** [Would you like to describe how your knowledge about your own health was acknowledged and utilised by the healthcare staff during your most last encounter]
**På vilket sätt bjöds du in att vara delaktig i planeringen av din vård?** [Can you describe how you were engaged in the decision-making process regarding your care plan?]
**2. Hur hände det?** [How did it happen]
**Kan du beskriva vad som föranledde att du sökte vård vid primärvården för din psykiska hälsa?** [Can you explain the circumstances or factors that prompted you to seek mental health support through primary health care?]
**Vill du beskriva dina strategier för att hantera din psykiska hälsa?** [Would you like to describe your strategies for managing your mental health?]
**3. Vad betyder händelsen för dig i din unika situation?** [What does the event mean to you in your unique situation?]
**Vilken är din erfarenhet av att söka vård för din psykiska hälsa generellt, och specifikt inom primärvården?** [What is your experience of seeking care for your mental health in general, and specifically within primary health care?]
**Vilket stöd skulle du önska vid kontakt med primärvården för din psykiska hälsa** [What kind of support would you wish for when seeking help from primary care for your mental health?]
**Hur upplever du mötet med Vårdpersonal inom Primärvården?** [How do you experience your encounters with healthcare professionals in primary health care?]
**Vilka strategier använder du sig av vid kontakt med vården för din psykiska hälsa?** [What strategies do you use when engaging with primary health services for your mental health?]

### Data analysis

In line with the descriptive phenomenology analysis by Giorgi [46] the analysis started by reading and re-reading the data and by listening to the interviews to hear the tone and the timbre of the voices, to get a sense of concrete descriptions of specific situations within the natural attitude of the phenomenon, i.e. encountering PHC for their MHPs. In line with Giorgi [46], the data was transformed by the authors’ adopted disciplinary attitude, which entailed a health science perspective and included a person-centered approach. This approach according to Ekman et al. [50] is based on the perspective of the person seeking care, a person with capabilities is to be viewed as an active partner in the decisions concerning his/her own care and treatment in collaboration with health professionals. The eidetic reduction was then performed, which was essential for the specific expressions used by the patients to describe the phenomenon from a health science perspective with a person-centered approach. The original meaning units, based on the spoken word of the person, were the experience-near terms that created the constituents, which were then used to manage the data. The specific constituents were identified and formed the four themes which explain the lived experience - the essence. The constituents were placed in a schematic diagram in Table 2 to gain an overview of the analysis. The interrelatedness of the constituents was analyzed to describe the casual aspect of the specific essence. The analysis involved all authors in the search for the essence (invariant meanings), the structures of meaning that describe the general structure of the lived experiences of patients with MHPs encountering PHC. The researchers aimed to understand meanings throughout the construction of the essence, which entailed a conscious act in order to untangle one’s own perception and experienced knowledge of the phenomenon and the sense in which the phenomenon was presented [46].

**Table 2.** Schematic diagram of the data analysis process.

Essence	Themes/Constituents	Codes- original meaning units
A desire to be embraced	To come from loneliness and vulnerability	*“Problems that cause problems may be small, they become huge”.*
		*“I was so vulnerable/ under the weather”.*
		*“I just wanted somewhere – someone- to talk about this”.*
	To sense mental health was viewed as problematic	*“I didn’t feel like I got any response that it was scary”.*
		*“Skip the talk and get down to business”.*
		*“It probably wasn’t MHPs in that way”.*
	To not be in control	*“Being bounced in many different directions”*
		*“The GP questioned me so hard”*
		*“Then off you go/nothing happens”.*
	To feel safe	*“Try to help me tell my story, who makes you feel safe”.*
		*“Right health professional”*
		*“The door was open, to feel better”.*

## Trustworthiness

To enhance the trustworthiness and quality of the study, the seven practical implications by Shorey & Ng [51] guided the performance of this descriptive phenomenological study (i.e. research objective, design, use of theoretical framework, sampling procedure, data collection, analysis, and presentation of findings). In addition, the Standards for Reporting Qualitative Research [SRQR] − 21 items were used to support the quality of the reporting of the qualitative data [52].

## Results

The socio-demographic characteristics of the patients are presented in Table 3. They varied in terms of age, gender, educational background, current employment, experience of MHPs, and latest professional encounter. For example, their experience of MHP varied from 1,5–18 years (median 12) and their age from 20 to 66 years (median 40).

**Table 3.** Socio-demographic characteristics of the patients.

Patients	Age*	Gender	Educational background	Current employment	Experience of MHP*	Professional Encounter
1	42	F	Vocational training	Self- Employed/ Sick leave	18	Physician
2	20	F	Upper Secondary School	Employed	5	Psychologist
3	40	M	University Degree	Employed	2	Counselor/Therapist
4	48	F	University Degree	Employed	14	Physician
5	26	F	University Degree	Student/ Employed	1,5	Psychologist
6	32	M	Upper Secondary School	Unemployed	10	Counselor/ Therapist
7	66	F	Upper Secondary School	Retired	12	Physician
8	31	M	University Degree	Employed	15	Physician
9	26	F	Upper Secondary School	Employed/ Sick leave	12	Physician
10	40	F	University Degree	Employed	5	Specialist Nurse
11	46	F	University Degree	Employed/Sick leave	12	Psychologist
Mean (range)	38 (20–66)				10 (1,5–18)	
Median	40				12	

* Years

### A desire to be embraced by health professionals

The essence of the lived experience of being a patient with MHPs in PHC was *a desire to be embraced* and the general structure of the essence was based on four themes: *To come from a place of loneliness and vulnerability*, *To sense mental health was viewed as problematic, To not be in control and To feel safe.* Being a patient with MHPs was described as having feelings of vulnerability. They were unsure of themselves and had signs and symptoms of MHPs. They found their MHPs difficult to comprehend which experience resulted in feelings of loneliness. It became difficult for them to describe their mental health to themselves and to their significant others or health professionals. When they found their mental health to become too difficult for them to manage on their own, they wished to receive professional support to ease their suffering, this was essential to them when reaching out to PHC services. The patients wanted and needed to engage in a dialogue about the signs and symptoms of their MHPs to make sense of their situation – their desire to be embraced. The patients described their situation as unbearable. Their suffering increased when patients encountered health professionals who were unable to support or understand them, i.e. embrace them, in *To sense mental health was viewed as problematic* and *To not be in control*. All they ever wanted was to be perceived as a person entitled to professionals’ support, i.e. *To feel safe.* It was critical to evoke the same emotional experience as one might have during a physical illness, in which they felt cared for and embraced by health professionals. The essence and its general structure based on these four themes is described in detail below.

### To come from a place of loneliness and vulnerability

The patients dealt with feelings of loneliness and having to manage the signs and symptoms of their MHPs by themselves before they sought help from the PHC. All of them described feelings of being in a vulnerable situation to the health professionals they met. One patient described it as being the lowest point in her/his life, while another described it as a situation where even the smallest setbacks became too large, and they felt it was difficult to manage on their own. Seeking support in this vulnerable situation was difficult for them. For some patients, it was the signs and symptoms of their MHPs that hindered them from reaching out to services, even though they knew they might have benefited from doing so.

I suffer from anxiety; I have anxiety problems. And it is that. I don’t deal with it. I become passive. But it is my anxiety that makes me not seek support. I should do it.

Informant 6

Some patients had support from their significant others, but there were some elements of their MHPs that they wanted to safeguard their significant others from. It was in some cases the significant others who had noticed that their mental health was deteriorating and becoming too difficult for them to manage and had prompted them to seek help at the PHC. The patients felt gratitude for having significant others who supported them and made sure they reached out for professional support.

Yes, my partner and I talk about a lot, as much as we can. However, there are some topics I avoid, keeping her from worrying. So, I do keep certain things to myself. As I mentioned, I’ve been trying to cope and remain hopeful about getting support, even if it’s delayed. I now have an appointment scheduled, along with sessions with both a psychologist and a private therapist.

Informant 3

The health professionals became the only way of receiving support for those patients who did not have anyone to talk to about their mental health. One patient was afraid of what would happen to them if their significant others found out about their mental health, since MHP was not something they talked about within their family.

I had no one to talk to. I did not have my family to talk to, so I felt the only place for me to turn to, was a counsellor or a psychologist.

Patient 2

Some patients described their very first encounter with PHC as entailing an initial barrier. They meant that as a patient with MHPs they had to build up energy prior to the encounter because of the loneliness and vulnerability they felt.

Being listened to and taken seriously, it’s different in different people’s lives in some way, but you still bring up something that is difficult for you, and that you almost feel a bit ashamed of it.

Patient 8

### To sense that mental health was viewed as problematic

The patients felt that the health professionals had an obligation to ask them about their mental health in every PHC encounter, even if they felt that the professional was not comfortable in managing their answer. Several patients sensed that mental health was talked about as being problematic, and that the professionals’ attitude affected the outcome of the encounter. As a patient, they knew from the start of the encounter whether or not they could talk about their MHPs. They described their fear of encountering a health professional whom they lacked a personal chemistry with. This was described as the professionals’ choice of words, the tone of their voice when initiating a dialogue about their mental health. The patients thus described encounters as ‘night’ and ‘day’ or as ‘right’ or ‘wrong’ i.e. receiving or not receiving support for their MHPs. Those encounters were dependent on the health professionals’ attitude and view on mental health. The patients felt an insecurity about which health professional or PHC provider they would encounter for their MHPs.

I think that if I need to contact them again about this, I’ll wait until the general practitioner I have, has time for an appointment in that case, I feel. I know which general practitioner I don’t want to go to. So, it feels a bit insecure to know that I don’t know whether I will get help there the next time.

Patient 4

Some patients recognised the various attitudes held by the health professionals as being limitations in their knowledge of mental health, which in turn was a result of the organisational restrictions of dealing with patients with MHPs. Patients described an underlying working culture within PHC where their problems should be able to be resolved rapidly and where the encounters were dependent on the health professionals’ attitude towards mental health.

We only had 30 minutes, to have a conversation for 30 minutes, to tell them everything in 30 minutes, it didn’t work for me

Patient 1

Most patients did not understand the health professionals’ attitude towards MHPs and mistrusted the health care system. Encountering a health professional, who could not understand them or was not interested in them, did not provide them with the opportunity to express their narrative, wishes, concerns, and worries. They felt there was an undertone of disbelief, a questioning of their perceived health and a feeling of being ridiculed as a patient in those encounters. This led the patients to rely on their own capacity to deal with their MHP and without support from the health professionals, they described feelings of being abandoned.

When I walked through the door the person said Adios Amigos like, what’s this, is this a joke or was I?

Patient 3

Most patients had encountered health professionals who were only interested in and prioritised their physical symptoms rather than the signs of their MHPs. The patients felt that they had to exaggerate their narrative in these encounters. They sensed that the signs and symptoms of MHP they showed were not investigated thoroughly by the health professionals. They experienced the ‘wrong’ encounter or an encounter where the health professional only focused on what was said, which in some cases had been limited due to their signs and symptoms. The patients believed that the professionals’ attitude contributed to their feeling of not being listening to, and thus did not grasp the whole picture of their situation. More specifically, the health professionals’ attitude and lack of knowledge was believed to limit their ability to show empathy towards the patient. This had negative consequences for the patients since those encounters were energy consuming and in turn challenged their abilities to manage their own health. The signs and symptoms of MHPs increased for some patients after such an encounter.

So, for us, it was like running a marathon and when I just reached the finish line, I was completely, completely exhausted. So, they [ed. health professional] just directed you further, “Now you must run 10 000-meter hurdles”. And what, I can’t bear it, I can’t, and it’s only them [ed. that can support you]. But go ahead, like, just run. That’s how it was for me.

Patient 11

### To not be in control

Feeling abandoned and not having the support from a health professional led to additional encounters for the patients in their search for professional support for their MHPs. Being a patient in these encounters and having to figure out how to get support felt as if they were being ‘bounced as a ball’ and ‘caught in a merry go-round’. They thus tried to console and manage their MHPs themselves, or with support from their significant others, until they encountered a health professional at the PHC who could support them. The patients experienced a lack of opportunities for care, support, and guidance from the health professionals to ease their suffering.

No, I thought I knew how to seek health care, but still I have bombarded them (PHC), with 1177 (national helpline service for health and illness), e-mail and phone calls and such. No, so I don’t have a good recipe.

Patient 3

They also described being offered limited care and treatment options. Cognitive behavioural therapy was described as ‘one size fits all’ regardless of the signs and symptoms they suffered from, and despite their patient history of having recently completed such treatment. The patients gave similar descriptions of being offered anti-depressive medication without being thoroughly assessed or being offered follow-ups. The responsibility to care for them as patients was non-existent. They felt it was up to them and felt powerless and were frustrated over how the PHC was organised.

All of Sweden seems to love cognitive behavioral therapy so much, but it’s never worked for me.

Patient 9

Feeling powerless also affected their relationship with the PHC as a first level of mental health service. The patients became used to being declined support or being put on hold (in a queue). Some explained that they needed to stop searching for support because of the energy it took from them being patients with MHPs. They were afraid of what would happen if their MHPs became too difficult to manage on their own, and the support from their significant others became particularly crucial. This situation raised concerns for themselves and for other patients with MHPs, a fear of not receiving support until it was too late (i.e. suicidal). Some patients were devastated over this experience since they thought they knew about how to receive care when being ill. The patients sensed and were told that they were not eligible for immediate attention of receiving support since they did not express suicidal thoughts. Some patients thus deliberately exaggerated their signs and symptoms as the only solution to receive support. They thought that some of their current problems could have been avoided, and their mental health would not have deteriorated if the patients had received support when they asked for and needed it. This experience had consequences for their view of themselves as being capable and they started to question themselves, they were perhaps not ill.

But it feels so strange to me, that a person who is already feeling so ill has to seek out a health professional who will take care of them. Plus, the waiting times are so extreme. so, in some way they want you to seek care from someone when you’re feeling ill. But you can’t get any appointment. Who guarantees that this person will continue to want to search for care or have the strengths to do so?

Patient 5

One patient described it as they knew that there was specialised MHS somewhere, but they felt they had no access to it. Feelings of rejection, neglection and not being worthy as a patient was present.

### To feel safe

The patients wanted to encounter a health professional who could support them, help them sort out their thoughts and make sense of these, and when these were identified enable a thorough assessment of their mental health. The thorough assessment should shed light on the signs and symptoms the patients had diffusely felt but had not been able to articulate. When the professional identified signs and symptoms that needed their further care or treatment, it was important for the patient to feel safe. Feeling safe was described as knowing that the health professionals had knowledge and a good attitude, and that the PHC organisation had a structure to deal with their symptoms. The health professional should then ask the ‘right’ questions and could offer them various forms of support. The symptoms were articulated by patients as they took an active part in the decision-making process concerning their care and were being taken seriously. The patients spoke of perceiving the reassurance of comfort and getting feelings of hope that led to a sense of relief when the health professional tried to support their narrative, and they felt that there was an alliance between them. The reciprocity in the encounter resulted in an active partnership where the patients contributed with their concerns, wishes and beliefs to what was needed to regain health. They felt safe, lucky, privileged, and grateful. Once the alliance was in place, it was described as if a door had opened towards better health.

No, but it was a nice feeling. A sense of relief that someone had finally listened and took the time to get to know my concerns and could help me get a solution. So, it was a relief and that alone shed that little light in the tunnel that you need, and the small hope like, that this I’ll be fine. That you had support from your general practitioner and the counsellor as well.

Patient 4

Encountering the ‘right’ health professional in a PHC organisation, which had the structure to encounter patients with MHPs, was described as entailing that their wishes and concerns were respected and that they were listened to and taken seriously. They became reassured that they could receive support to ease their suffering and that there was support for them to access it when they needed it. The support given was based on what the patients described as a thorough assessment and the treatment and care was tailored to fit their needs. Some patients thus described feelings of gratitude towards the care they received for their MHPs.

On the same day that I’d called, I got to talk to a psychiatric nurse and was booked for a clinical assessment with a general practitioner and a therapist. There were others that…. It felt, both then and now, quite uncomplicated. There was a clear structure.

Patient 10

## Discussion

This study contributed with knowledge about the lived experience of a person with MHPs who encountered PHC as patients when in need of care. Their lived experience was dominated by a *desire to be embraced* by the health professional they encountered within the PHC. Being person with MHPs was coming from a place of loneliness and vulnerability before becoming a patient of the PHC. They described it as feeling doubtful and insecure about reaching out for support, especially since they had experienced public and self-stigma in previous encounters. Presenting themselves to the PHC thus became ambiguous; they needed support to be able to cope with their signs and symptoms of MHP but were also afraid of being exposed to stigma by professionals and/or a PHC without the organisational structure to enable patients being embraced. This initial barrier is also described by Tunks et al. [53], where the patients’ view of PHC creates a barrier for them to reach out for support. Moreover, a PHC without the organisational structure to deal with them as patients created frustration. This finding is reflected in the results in qualitative studies of health professionals working in a PHC, who experienced little or no capacity to deal with a person encountering PHC for their MHPs [22,54,55]. The lack of organisational structure in the present study was described as a lack of structure and guidance in the encounter from the health professional, in comparison to the patients’ descriptions of encounters focusing on physical symptoms and signs. They thus doubted whether their main concern for their health was a real concern for the health professional and the PHC.

To access and maintain PHC for people with MHPs has been described as challenging and difficult [13,14]. A lack of knowledge about mental health and the diversity in the PHC organisation of how to deal with patients with MHPs created a disbelief towards PHC as being a first level of mental health service. In addition, some encounters resulted in a decrease of health and hindered patients in their recovery process. The importance of the health professional initiating a dialogue about mental health during the encounter was thus described as manifest. The encounter with a health professional, who gave them the opportunity to sort out their narrative, was described as someone who listened to them, viewed them as a person with capabilities with a non-judgemental attitude and took them seriously. This is consistent with a person-centred approach, as described by Ekman [50,56] and Kristensson-Uggla [57]. In addition, the result of this study complements the results public health nurses encounter patients with MHPs in PHC [22,55,58,59]. Their attitude and knowledge about mental health as well as the lack of organisational structure and resources for mental health encounters hinders them from providing accessible and integrated mental health services in PHC.

Being that the PHC is the first level of mental health service in Sweden and has an important remit to deliver equal health care to the population [60], various attempts to integrate mental health services into the PHC have been made. However, the integration across PHC varies [25], as reflected by the lived experience of patients from our study and from how public health nurses’ experienced encountering patients with MHPs [22]. A fragmented mental health service was a common experience and there was a lack of continuity of care. The integration of care managers in PHC setting may be one solution to solve some of these problems [18,19]. Yet, the mental health interventions provided was limited to brief visits, cognitive behavioural therapy, and antidepressive medication. The result is in line with WHO’s description of today’s mental health services, based on a traditional patient-centered care approach with a focus on diagnosis, medication, and symptom reduction [1]. This limits the opportunities for recovering a meaningful life, in accordance with a person-centred care [50,56,57]. While the process of integrating mental health services in PHC is ongoing with, for example, the Collaborative Care Model [17] and the Integrating Behavioural Health and Primary Care [16] helping to lead the way, specialist mental health services currently aim for integrating services based on the patients’ mental health needs. This entails that municipal health care (a part of the PHC) and social services are integrated to provide more opportunities of support and care patients with MHP. The lived experiences of patients who participate in the Flexible Assertive Community Treatment (FACT) model, as described in a study by Borgh et al. [61], show that care and support are facilitated by genuine relationships. This relationship is built on experiences of equal partnership, feelings of being seen, and using tailored care and support ‘when and where they need it’, care that allows for rebuilding everyday life. Equal partnership also contributes to health professionals experiencing a lower level of job-strain and stress and contributed to a higher quality of care for the patients [62]. Important elements in the FACT model that contribute to its success are the exposure to the narratives of the patient and/or their significant other. Within this co-production of care from a person-centred approach, the patient is acknowledged as an active team member. Being exposed to the narratives of the patient with a lived experience can reduce the stigmatizing attitude of the health professionals and their empathy can increase [35,63]. It is therefore essential to include a dialogue about lived experience of MHP in the further development of PHC. In addition, it is critical to address health professionals’ attitude and knowledge about mental health in their workplace when starting the dialogue in the encounter. A high level of mental health literacy among professionals is critical when patients present themselves with a health concern, which has consequences for the patients’ health process and future contacts with health care providers [64], as confirmed by our result. It is therefore essential to include patients’ lived experiences as a part of the experiential knowlegde concept to inform future interventions and initiatives to empower patients and improve care [65].

## Trustworthiness and limitations

There are some limitations that should be acknowledged when interpreting the results of this study. Firstly, the researchers were novices in the use of a descriptive phenomenology design. Being novices led to an in-depth study of phenomenology as a method, using a critical approach during our discussion and in our interpretation of Husserl’s philosophy. To enhance quality and trustworthiness of results, the authors own experiences and preunderstanding of the phenomenon is presented. To strengthen the quality and trustworthiness of the study, we constructed an interview guide inspired by methodology articles of the field [46,48,49]. The interview guide may potentially have limited the participants’ spontaneous descriptions of the phenomenon as it is presented to them [66], but on the other hand it also supported the authors (EN, SJ) in gaining an in-depth (rich) description of the phenomenon, thus increasing the study’s dependability. Secondly, we utilized a purposive sampling procedure that probably had a natural influence of the convenience sampling during data collection process. This may have restricted a full gain of the variation of descriptions of the phenomenon [67] and may thus limit the transferability of the study. Furthermore, when including potential participants from the age of 16 years and older, using an informative ad in social media, this may have limited older patients to participate. Using social media as a platform has shown to limit the involvement of older populations [68]. Therefore, we also included recruitment of potential participants with posters in PHC settings. Thirdly, our interviews were held *via* LU-Zoom platform, digital interviewing has shown to be an alternative, high-quality data source when aiming to gain a variation of experiences not constrained to the whereabouts of the researcher [66]. However, using LU-Zoom as a platform had the potential to limit participation of those participants who are not being comfortable with being digitally interviewed, which may also have limited the study’s transferability. Fourthly, according to Giorgi [46], phenomenology requires at least three study participants to perform the data analysis, since the data analysis process requires in-depth richness. For methodology stringency, all participants who met the inclusion criterions were included. The socio-demographic description of the participants was believed to add value to the participants lived experiences of encountering PHC. In qualitative research, such socio-demographic information may increase the transferability of the study to other settings [69]. A triangulation procedure between the researchers during the data collection, data analysis and writing of the results, was constant. The authors returned to the raw data when the results were presented and the ongoing discussion between the researchers has increased the credibility and conformability of the results. By using the qualitative guidelines [51,52] the study’s credibility was supported by enhancing quality and trustworthiness. The result is judged transferable to similar contexts of being a person with MHPs in need of first level of health care services.

## Conclusion

The essence of the lived experience of being a patient with MHS was a desire to be embraced in the encounter with a health professional was to receive adequate support and guidance based on their signs and symptoms of MHPs. Further, the result indicates that the lived experience for patients with MHPs is not adequately accommodated when seeking help in PHC. The patients face challenges due to their mental health and the experiences of encountering health professionals with a lack of knowledge and negative attitude could deteriorate their health. In the Good Quality and Local Healthcare – a Primary Care Reform [60], it is stated that the transformation from a patient-centered to a person-centered PHC is crucial. The first step is to acknowledge patients as experts concerning their life situation, and as a core element in a person-centered care [50,56]. It is therefore essential for further research that patients’ experiential knowledge is included in order to inform future research and clinical practice.

## Data Availability

The datasets generated and/or analysis during the current study are not publicly available due to ethical considerations from the participants’ point of view but are available from the corresponding author on reasonable request.
